# Supplementary figures and images for: The Alk receptor tyrosine kinase regulates Sparkly, a novel activity regulating neuropeptide precursor in the Drosophila central nervous system
Source: eLife. 2024 Jun 21;12:RP88985. doi: 10.7554/eLife.88985 (PMC11196111; doi:10.7554/eLife.88985)

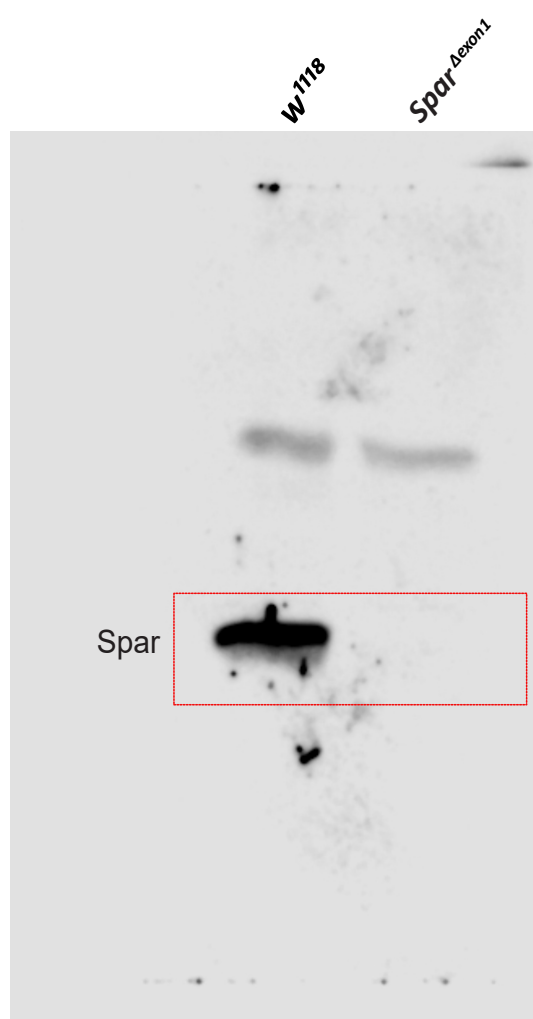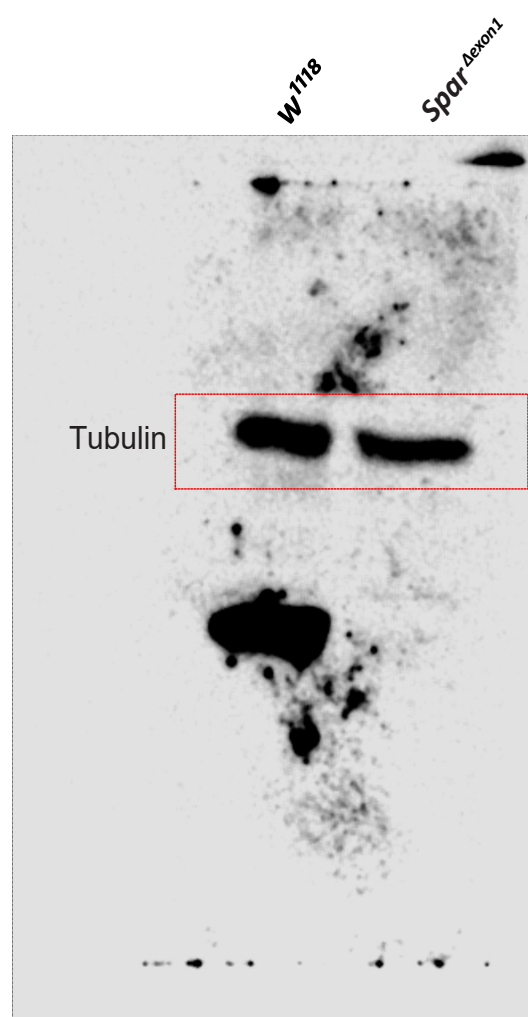

Supplement: Figure 7—source data 1. [file elife-88985-fig7-data1.zip › full blot marked.pdf]

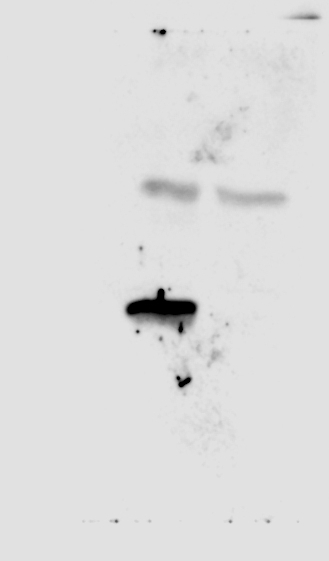

Supplement: Figure 7—source data 1. [file elife-88985-fig7-data1.zip › Raw file Spar_1.jpg]

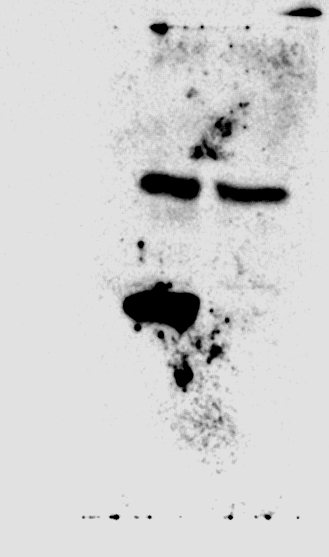

Supplement: Figure 7—source data 1. [file elife-88985-fig7-data1.zip › Raw file tublin_2_long exposure.jpg]
